# Understanding preferences of stroke survivors for feedback provision about functional movement behavior from wearable sensors: a mixed-methods study

**DOI:** 10.21203/rs.3.rs-2789807/v1

**Published:** 2023-04-13

**Authors:** Marika Demers, Amelia Cain, Lauri Bishop, Tanisha Gunby, Justin B Rowe, Daniel Zondervan, Carolee J Winstein

**Affiliations:** University of Montreal; University of California, Los Angeles; University of California, Los Angeles; University of California, Los Angeles; Flint Rehabilitation Devices (United States); Flint Rehabilitation Devices (United States); University of California, Los Angeles

**Keywords:** Wearable electronic devices, stroke, rehabilitation, feedback, mobility, upper extremity, behavior change

## Abstract

**Background:**

In stroke rehabilitation, wearable technology can be used as an intervention modality by providing timely, meaningful feedback on motor performance. Stroke survivors’ preferences may offer a unique perspective on what metrics are intuitive, actionable, and meaningful to change behavior. However, few studies have identified feedback preferences from stroke survivors. This project aims to determine stroke survivors’ satisfaction with feedback from wearable sensors (both mobility and arm/hand use) and to identify preferences for feedback type and delivery schedule.

**Methods:**

A sample of 30 chronic stroke survivors wore a multi-sensor system in the natural environment over a 1-week monitoring period. The sensor system captured time in active movement of each arm, arm use ratio, step counts and stance time symmetry. Using the data from the monitoring period, participants were presented with a movement report with visual displays of quantitative and qualitative feedback. A survey and qualitative interview were used to assess ease of understanding, actionability and components of feedback that users found most meaningful to drive lasting behavior change.

**Results:**

Arm/hand use and mobility sensor-derived feedback metrics were easy to understand and actionable. The preferred metric to encourage arm/hand use was the hourly arm use bar plot, and similarly the preferred metric to encourage mobility was the hourly steps bar plot, which were each ranked as top choice by 40% of participants. Participants perceived that quantitative (i.e., step counts) and qualitative (i.e., stance time symmetry) mobility metrics provided complementary information. Three main themes emerged from the qualitative analysis: 1) Motivation for behavior change, 2) Real-time feedback based on individual goals, and 3) Value of experienced clinicians for prescription and accountability. Participants stressed the importance of having feedback tailored to their own personalized goals and receiving guidance from clinicians on strategies to progress and increase functional movement behavior in the unsupervised home and community setting.

**Conclusion:**

The resulting technology has the potential to integrate engineering and personalized rehabilitation to maximize participation in meaningful life activities outside clinical settings in a less structured environment—one where stroke survivors live their lives.

## 1. Background

The rapidly changing demographics in the United States have led to an increasing need for solutions to promote rehabilitation outside clinical settings [1]. Increasingly, wearable sensors have been recognized as a potential response to this growing need [2, 3], due to their capacity to capture functional movement behavior (both locomotion and arm and hand use) and provide timely, meaningful feedback to the user about motor performance. Understanding how stroke survivors perform in the home and community, within its unstructured and often unpredictable context, can be useful to guide personalized clinical interventions [4]. Wearable technology can be used as both a contextually relevant assessment method and simultaneously an intervention modality to facilitate behavior change through provision of direct feedback to the user about their activity [5, 6]. Mobile applications can often be combined with wearable sensors to offer reminders, encouragement, education, or messages to indicate how many more steps/minutes of activity are needed to achieve personal activity goals. The benefit of wearable technology (i.e., digital therapeutic) for stroke rehabilitation include their unobtrusive nature (i.e. data can be captured without hindering everyday activities) and most importantly the possibility to deliver therapy in the context of an individuals’ everyday life [7, 8].

Feedback provided by wearable sensors can encourage health-promoting behaviors for stroke survivors, such as physical activity and the choice to engage functional behaviors (e.g., upper limb use, mobility activities) [2]. For stroke survivors, strong evidence supports the provision of extrinsic feedback to elicit motor learning processes and improve motor recovery [9]. There is growing evidence that extrinsic feedback can positively influence motivation, self-efficacy, and compliance [10, 11]. Feedback provided through wearable sensors may include objective measures of activity (e.g., step or movement count, sedentary time, time in active movement for each arm), graphs of daily activity, or reminders/encouragement towards activity goals (e.g., encouragement to close your activity ring) [2, 12]. Feedback frequency (e.g., daily, on-demand, when someone is inactive for a prolonged period), and modality (e.g., auditory, haptic, or visual) can vary from one system to another. However, while these attractive features of a wearable sensing system are all possible, it does not follow that they are all inevitable! On the contrary, a wearable sensing system must be carefully designed to foster these desirable features and attributes. Moreover, due to the many options of feedback types and modalities, it can be challenging to make informed design decisions on what may be best to drive behavior change for an individual stroke survivor with their unique demographic, psychosocial and clinical profile.

User centered design is critical for considering and addressing the clinical and personal needs of the target population [13]. To date, few studies have identified feedback preferences from stroke survivors. In a stakeholder survey of a wearable activity monitor for upper limb recovery, 35% of stroke survivors preferred feedback via a combination of vibration and sound whereas 29% preferred a visual message [14]. In a systematic review on wearable sensors for upper limb rehabilitation across different health conditions, visual display was the most common way to provide feedback. Most systems attempt (either intentionally or intuitively) to indicate progress towards an understood skill or goal, an approach known in the field of psychology as Knowledge of Results [12]. To promote skilled motor recovery and maximize community participation, wearable sensors need to provide actionable feedback about movement quantity and quality that users can understand and implement to motivate activity and change behavior. If the ultimate goal is to personalize the digital therapeutic, it is equally important to understand the feedback preferences (type, delivery schedule, and meaningfulness) from the user; in this case, community-dwelling stroke survivors.

This project aims to: 1) determine stroke survivors’ satisfaction with feedback from wearable sensors (both arm/hand use and mobility), 2) identify stroke survivors’ preferences for feedback type and delivery schedule that has the potential to drive health-supporting behavior change.

## 2. Methods

### 2.1. Study design:

This study used a mixed-methods convergent design [15] to identify feedback preferences of stroke survivors. This study is part of a larger project aimed to develop a data-driven and clinically informed behavioral intervention strategy for a wearable sensor system that uses actionable quantitative and qualitative feedback to maximize physical function after stroke [16, 17]. We used a quantitative survey to identify ease of understanding and actionability of each feedback metric, whereas qualitative semi-structured interviews allowed a deeper understanding of users’ feedback preferences needed to drive lasting behavior changes. The collection of qualitative and quantitative data allowed a rich, comprehensive, and actionable dataset to inform future development of a user-centered digital therapeutic intervention.

### 2.2. Participants:

Community-dwelling stroke survivors aged > 18 years old, independent with home or community ambulation (with or without supervision) and fluent in English were included. We excluded participants with unilateral spatial neglect, severe cognitive or language impairments or other medical condition that may interfere with participation. Communication facilitation strategies were used to allow participants with mild-moderate aphasia to participate. The sample size was based on the concept of data saturation for the semi-structured interview data. It was estimated that a sample of 25–30 stroke survivors would be sufficient to reach saturation. Stroke survivors were recruited from the IRB-approved Registry for Healthy Aging Database (RARE). We also recruited a convenience sample of ten age-matched non-disabled neurotypical participants through contacts of the research team and email advertisement. All participants were fully informed of the procedures involved and provided informed consent. Study procedures were approved by the Institutional Review Board at the University of Southern California (HS 19–00984 and HS 20 − 00015).

### 2.3. Procedures:

Stroke survivors wore a wearable sensor system (MiGo, Flint Rehabilitation Devices, Irvine, CA) for a one-week, whereas neurotypical participants wore the system for 24h. The MiGo system consists of two wristwatches and a sensor strapped around each ankle. While the MiGo has feedback capability, feedback from the wristwatch was intentionally disabled during the monitoring period. To facilitate donning/doffing, the wristband on the less affected side was replaced by an elastic strap. Prior to the monitoring period, the research team collected demographic and clinical data and provided information on how to don/doff and charge the sensors. Participants were instructed to wear the sensors for 12h/day and continue their typical activities.

#### 2.3.1: Data acquisition from wearable sensors:

During the monitoring period, the watches logged upper limb active time and the ankle sensors logged both step counts and the average percent of the gait cycle spent in the stance phase. Logs were saved every quarter hour. For upper limb active time and step count, logs recorded the current daily total. For stance percent, the estimate was reset every 15 minutes and a new estimate was collected in every log period.

Participants were given a cellular gateway (Tenovi Health, Irvine, CA) that automatically retrieved logs from the MiGo devices every 3 hours and conveyed them to a secure, HIPAA compliant server accessible only to members of the research team.

**Figure 1:**
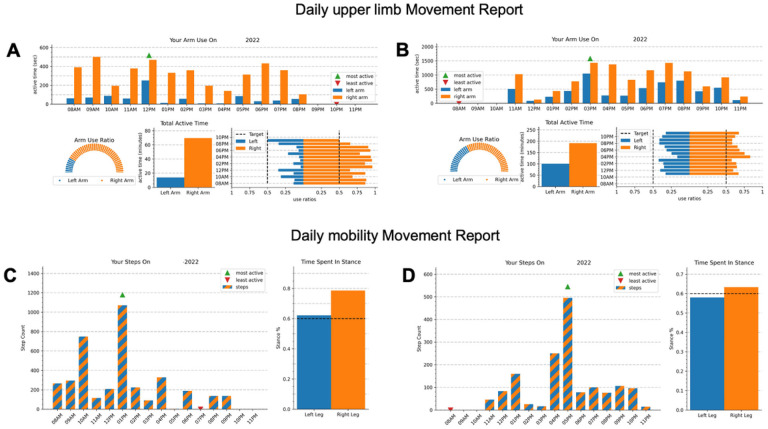
Example of daily Movement reports: Example of two daily movement reports generated after a one-week monitoring period for two representative participants with a right hemispheric stroke: one with severe motor impairments (A and C; Fugl-Meyer Assessment score of 28/66, Functional Ambulation Category of 3 – supervision) and one with mild motor impairments (B and D; Fugl-Meyer Assessment score of 64/66, Functional Ambulation Category of 5 – independent). Left (paretic) arm/leg movements are in blue (dark), and Right (less paretic) arm/leg movements are in orange (light). A and B: The top graphs represent the daily upper limb, movement report with an hourly arm use bar graph (top), a dial plot (bottom left), a daily active time bar graph (bottom center) and an arm use ratio horizontal bar plot (bottom right). C and D: The bottom graphs represent the daily mobility Movement report with the hourly step counts on the left and the time spent in stance on the right.

After the monitoring period, participants returned the equipment and met with a member of the research team in-person or remotely using the Zoom Meetings platform (Zoom Video Communications, San Jose, CA). Before the meetings, the team used a custom-built client application (app) to request data from the server and process it to generate individualized movement reports. (Fig. 1). For the upper limb reports, the client would request all the available active time logs for a given participant on a given day. The difference from one log to the next was taken to determine the amount of active time in each 15-minute bin. For each bin we computed the ratios of the left and right arm active times to the total combined active time to obtain the normalized use ratio. For each day in the dataset, we resampled the data into 1-hour bins and generated a report showing the hourly active time, the total active time for the day, the normalized active time ratio for the day, and the hourly active time ratio. We resampled the data into one-day bins and generated a report for the entire week showing the total active time for each day, the average normalized use ratio for the week. In the weekly report, we also included a 2D histogram analogous to those presented by Lang et al. [18] representing all 15-minute bins from the week with the normalized use ratio on the x-axis and combined (left and right) active time on the y-axis. Except for the 2D histogram, all plots were designed to emphasize ease of interpretation by end users. Data from the left and right sensors were color coded as blue and orange respectively and whenever possible data from the left and right sensors were shown on corresponding sides of the plots. Because the plots were intended for interpretation by the end users, more complicated features like error bars that might normally increase depth of interpretation were omitted in favor of simplicity.

The client app generated similar reports for mobility. For each day, it created a report showing the hourly step count and the average stance percent estimates for both legs. It also generates a report for the week showing the daily step counts and the stance percent estimates for each day. Stance percent estimates from logs containing less than 25 steps in a 15-minute period were excluded from the reports.

In the follow-up visit, participants were oriented to each graph on the movement report and reviewed the report with a research therapist. A 24-hour movement report from an age- and gender-matched neurotypical participant was also presented as a comparison to assist participants in the interpretation of their individualized report and to begin a discussion about their goals. Once the participant understood the movement report, they were asked to complete the movement report survey. We then conducted a 30-minute semi-structured interview about feedback preferences with each participant.

#### 2.3.2. Quantitative:

Participants completed the Montreal Cognitive Assessment [19] and the National Institutes of Health Stroke Scale [20], as descriptive measures of cognitive function and stroke severity, respectively. Arm and hand motor impairments were characterized using the Fugl-Meyer Assessment upper extremity (FMA-UE; severe: 0–28, moderate: 29–42, mild: 43–66) [21]. Walking ability was categorized using the Functional Ambulation Category (FAC; 3: walk with supervision; 4: walk independently on ground level; 5: walk independently anywhere) [22] and the 10-meter walk test [23].

Stroke survivors answered a movement report survey, which consisted of 19 visual analogue scales on a 10-cm line representing their ranking. They graded their responses based on ease of understanding (from very difficult to very easy) and movement encouragement (strongly disagree to strongly agree) for all arm/hand and mobility metrics. Higher scores indicated greater satisfaction. Participants were also asked to rank what they would prefer to see in the future by numbering each metric from 1 (highest) to 4 (lowest). Scores were derived from the visual analogue scales by measuring with a ruler the distance in millimeters between the participant’s mark and the 0.

#### 2.3.3. Qualitative:

Semi-structured interviews were conducted in a closed room and were audio-recorded to permit verification and clarification of content. Interview questions were based on an interview guide and structured to identify feedback preferences, usefulness of the movement report, and how feedback would be used in daily activities (see the Interview Guide in Additional file 1). The main ideas expressed during the interview were summarized at the end of each interview for member checking.

### 2.4. Data analysis:

#### 2.4.1. Quantitative:

Descriptive statistics were used to characterize our sample and report understanding and movement encouragement of each feedback metric. The means and standard deviations of the scores were determined from the visual analogue scales.

#### 2.4.2. Qualitative:

Interviews were transcribed verbatim. Two independent researchers (MD and AC) used inductive thematic content analysis to interpret meaning from the context of textual data [24]. The Braun & Clark framework [25] was followed and a detailed code book listing all the codes and definitions was developed to facilitate thematic content analysis. The initial coding was initiated after the first five participants were collected and performed until data saturation was reached. Data saturation was determined when no new codes emerged, and the ideas were repeated among participants. Repeated discussions occurred between the two coders to clarify interpretation of the data. A third reviewer (CJW) participated in the discussion related to refining the themes and data interpretation. Qualitative data analysis was performed using the NVivo software (QSR International Pty Ltd, Melbourne, Australia). Any disagreement was resolved by discussion and an audit trail was kept for the rationale behind every decision.

#### 2.4.3. Integration of qualitative and quantitative data:

Quantitative and qualitative data about feedback preferences were integrated once the data analysis was completed to identify metrics that are intuitive, actionable, and meaningful to drive positive behavior change. Qualitative data provided more nuances as to why one metric was preferred over another.

## 3. Results

A total of 30 chronic stroke survivors (mean 7.6 years post-stroke) took part in this study (see Table 1 for participant characteristics). The mean and standard deviation age was 58.6 ± 13.1 years. Participants had mild-severe motor impairments, with an average FMA-UE score of 41.2/66. Most participants were independent in ambulation (60% with an FAC of 5) and the average self-paced gait speed was 0.75 ± 0.40 m/s. Here we are reporting quantitative and qualitative results from surveys and interviews evaluating the feedback preferences of the participants.

### 3.1. Quantitative

**Figure 3:**
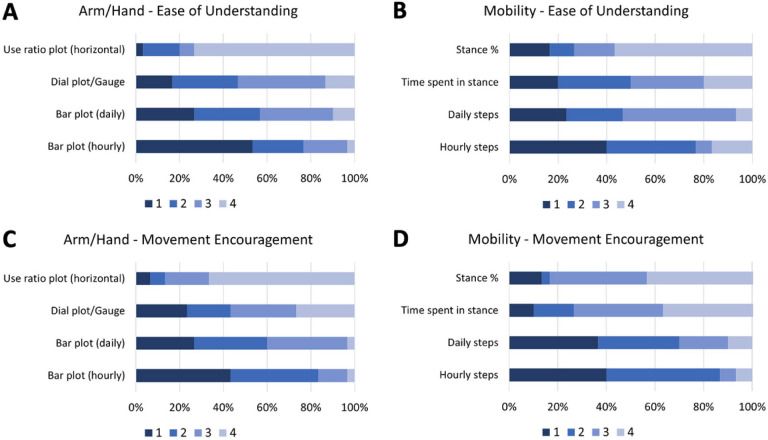
Ranking preferences for each feedback metric: Ranking preferences (1 is the most preferred and 4 is the least preferred) for ease of understanding (A and B) and movement encouragement (C and D) for arm/hand (A and C) and mobility (B and D) metrics.

All four arm/hand metrics were easy to understand (score range: 8.0–8.7/10) and three encouraged participants to move their arm/hand more (7.5–7.9). However, the ‘Use Ratios 2D histograms’ were less actionable (6.8 ± 2.4). For mobility, all four metrics were easy to understand (8.6–8.9) and actionable (7.8–8.3). When participants were asked to rank preferences for arm/hand movement encouragement, the ‘Hourly Arm Use Bar Plot’ (Fig. 1A and B) was ranked the highest by 40.0% of participants followed by the ‘Daily Arm Use Bar Plot’ (Fig. 1, A and B; 26.7%) and the ‘Arm Use Ratio’ (Fig. 1A and B; 23.3%). For Mobility, ‘Hourly Steps Bar Plot’ (Fig. 1C and D) was preferred by 40.0% of participants, followed by ‘Daily Steps Bar Plot’ (36.7%) and ‘Stance % This Week’ (13.3%; see Fig. 3 for ranking preferences).

### 3.2. Qualitative

Three main themes emerged from the qualitative data analysis: 1) Motivation for behavior change, 2) Need for real-time feedback based on individual goals and 3) Value of guidance from experienced clinicians for prescription and accountability. In the next section, each emergent theme is summarized along with exemplar quotes from each individual participants (i.e., S#).

#### 3.2.1 Theme 1: Motivation for behavior change:

This theme encompassed the perceived benefits of the wearable technology to encourage healthier movement behavior. The wearable technology was perceived as motivating and as a complement to rehabilitation care. Participants reported that the feedback metrics were easy to understand after they were oriented to each metric, with mobility metrics being easier to understand than the arm/hand metrics.

S44: ‘The report is very clear and easy to understand.’
S17: ‘[The mobility graphs] were more understanding than the arm graphs.’

While the feedback capability was deactivated during the data collection period, motivation for behavior change and the prospect to track one’s progress was identified as a potential benefit of the wearable technology. Participants were encouraged to see their motor performance on the movement report. For some, it validated their own performance assessment, while others were surprised about their daily performance and indicated that it provided hope for recovery.

S03: ‘Knowing how much movement you do during the day and the week is very helpful and kind of gets you to want to do a little bit more. [The sensors] are a tool to find out how you’re doing. That’s motivation.’
S12: ‘I liked the graphs. They were very encouraging because I didn’t know I was moving that much.’
S09: ‘I didn’t even know [my paretic arm] was moving that much. I’m very happy.’

Participants with severe motor impairments perceived that they were already using their paretic upper limb at their maximum capacity. They indicated that receiving feedback would have limited usefulness, as they did not have enough function of their paretic limb to use it more. They did not intuit a way that they could act on the provided feedback. Improving capacity or problem-solving with participants on how the more affected arm can be incorporated in daily activities may be more useful for participants with severe motor impairments.

S18: ‘I don’t think any report about using my arm would do anything, because I use my arm as much as I can.’

#### 3.2.2 Theme 2 - Real-time feedback based on individual goals:

The lack of real-time feedback delivery was identified by many participants as a limitation of our study design, as participants preferred to get something out of the wearable technology. Participants discussed the relevance to receive personalized feedback when needed (i.e., self-controlled feedback). For some participants, a daily summary sent by email was judged sufficient, while most preferred to receive feedback in real-time, directly on the watch or a companion app.

S22: ‘I liked what I saw. I would just want it sooner, so at the end of the day, you could see: “Oh, I haven’t moved”.’
S37: ‘An app would be really good, because I usually always have my phone with me. I could kind of look and say ‘Oh, you didn’t move too much today. Maybe, you need to move a little bit more’.’

Participants valued personalized goals. Most expressed wanting to receive feedback related to the accomplishment of their own goals and embed new goals as they progress.

S06: ‘For me, it would be more like, convincing me more. Like you’re giving me a certain [number] of steps that I will do. It challenges me.’
S05: ‘I set myself goals that I do 12,000 steps a day, and I have to reach that goal.’

#### 3.2.3 Theme 3 - Value of experienced clinicians for prescription and accountability:

Despite the potential of wearable technology to provide useful feedback directly to its users, participants reported that they would value meeting with experienced clinicians to help them set goals, to guide their progression and to help them problem solve ways to improve their upper extremity use habits. Many stroke survivors also reported that having goals and frequent meetings with therapists to monitor behavior was ideal to promote behavior change, foster accountability and offer strategies to facilitate movement during daily activities.

S12: ‘[Wearable technology] would be huge along with what I received in therapy. This would be huge. That would be a powerful tool in rehabilitation.’
S17: ‘I have to ask [therapists] questions. It’d be nice if I can meet with somebody.’
S31: ‘The therapist would actually see how you’re walking and stuff and give you tips.’

### 3.3. Quantitative and qualitative

While stroke survivors valued feedback on motor performance (i.e., time in active movement, arm use ratio, step counts or stance time symmetry), a single metric was not consistently identified in the surveys or interviews as being best for movement encouragement. For mobility, both quantitative and qualitative metrics were seen as useful and provided complementary information about gait. Visual feedback was appreciated by participants and a few reported that it could be enhanced by providing haptic or auditory feedback when a goal was met or to remind the participant to move. However, there was no consensus on whether people preferred positive (e.g., you’re doing great!) or negative (e.g., you haven’t moved in a while) feedback.

## 4. Discussion

This study aimed to identify stroke survivors’ preferences for feedback metrics. Multiple sensor-derived metrics were identified as easy to understand and actionable to encourage arm/hand movements and mobility. Participants perceived that real-time feedback and daily summaries may be useful to induce behavior change. Stroke survivors stressed the importance to provide feedback in the context of individual goals to motivate engagement. Meeting with experienced clinicians in conjunction with wearable technology were valued to foster accountability and offer strategies to facilitate movement during daily activities. To our knowledge, this is the first study to incorporate both qualitative and quantitative methods to inform feedback preferences design of wearable technology.

Individual preferences are important to consider in the design of an intervention [13]. Our results demonstrate that many different visual plots may be intuitive and actionable. Since data from wearable technology can be used by both stroke survivors and clinicians, the balance between ease of understanding for stroke survivors and relevant information to guide individualized treatment is delicate. A companion app for stroke survivors and clinicians could be a viable solution to allow users to access their preferred visual plot with advanced options for clinicians. Apps offer many possibilities to encourage health-promoting behavior. App features and characteristics may include activity data visualization, self-monitoring, personalized goal setting, general or tailored education, reminders, and social support via social media or a community forum [26]. Simple metrics such as percentage of paretic arm use, step counts or stance time symmetry, identified as being actionable to stroke survivors, could be displayed directly on an activity watch and accessed when needed.

Consistent with the findings from Wang et al. [12], visual displays were valuable and could be combined with auditory or haptic feedback to draw attention to specific behavior (i.e., long periods of inactivity or goal achievement). Self-controlled feedback (i.e., ability to access information when desired) was preferred by our participants. In the motor learning literature, evidence supports to use of self-controlled feedback to enhance motor learning [11]. Self-controlled feedback may encourage intrinsic motivation, support for autonomy and competence, as the individual takes charge of their own learning [11, 27, 28]. Our results also highlight the need to offer feedback in the context of individual goals. Goal setting is an integral part of stroke rehabilitation [29, 30]. Specifically, individualized goal setting may enhance motivation, adherence and autonomy, positively influence stroke survivors’ perceptions of participation, and improve recovery and performance [31]. Consistent with the growing efforts for precision medicine in healthcare [32–35], personalized approaches to stroke recovery are needed to account for the heterogeneity of impairments and disability after stroke.

While wearable technology enables stroke survivors to track their progress, feedback alone was not judged as sufficient to drive behavior change. Interventions should be carefully designed due to the complexity of behavior change. Simple feedback from wearable sensors alone (e.g., step counts) is not sufficient to change physical activity behavior of community-dwelling stroke survivors [37–39]. This emphasizes the importance to develop interventions using wearable technology that are grounded in strong theoretical foundations. Wearable technology should be integrated in clinical care to augment, not replace clinicians. Clinicians play a crucial role to offer personalized education and work collaboratively with stroke survivors to identify strategies to encourage movement performance and prescribe exercises. Patient-therapist interaction and therapeutic alliance were shown to increase treatment adherence and satisfaction, and is directly linked to positive rehabilitation outcomes [36].

Finally, wearable technology may not be for everyone. From our results, stroke survivors with severe arm motor impairments may not benefit to the same extent from real-time feedback about paretic arm use. Previous work indicates that there might be a minimal threshold in motor capacity for stroke survivors to incorporate their paretic arm in daily activities [40–43]. For example, Chen et al. [42] demonstrated that a score of approximately 50 on the FMA-UE may be a significant cut-off point for engaging unimanual paretic movements in the unsupervised home environment. The predominant UE spontaneous movements that are seen in patients with FMA-UE lower than 50 are bimanual tasks for which the paretic limb acts as an assist to the less-impaired side [42]. The characteristics of stroke survivors most likely to benefit from interventions using wearable technology remain to be empirically tested.

### 4.1. Limitations

A limitation to this study is that the participants were recruited from a database of survivors of stroke who volunteered to participate in research, limiting the generalizability of the results to the general stroke populations. These individuals may be more motivated to use the MiGo sensors and may respond more favorably to them. Therefore, we cannot exclude the possibility of a social desirability bias. We also noticed a discrepancy between the visual analogue scale and the ranking (i.e., some participants gave high scores on a given plot on the visual analogue scale but ranked them low in the ranking preferences). This may show a lack of understanding of the movement report survey. Despite these limitations, the qualitative methods that are used during the study provided a more in-depth understanding of the participants’ preferences of the metrics on the movement report. Moreover, we limited the number of visual plots we presented to participants to avoid exhausting them or biasing their interpretation of other plots. This limited our ability to test more complex plots that could be useful to clinicians. It should also be noted that the feedback preferences identified by stroke survivors may not directly translate to improved motor performance in daily activities, as we did not provide real-time feedback to our participants. In future studies, stance percent should be averaged over longer time intervals and samples from short bouts of steps should be excluded from the averages.

## 5. Conclusions

Using the Movement report as a starting place, we identified that stroke survivors found sensor-derived metrics intuitive and actionable to incorporate their paretic arm/hand into daily activities and increase walking behavior (amount and symmetry). Our findings underscore the importance of using wearable sensors along with personalized goals to motivate engagement outside the clinical environment. Wearable technology could be introduced earlier in the rehabilitation process as a complement to clinical care, as therapist-patient interaction is crucial to foster accountability and motivation from the beginning. This work will establish the groundwork for the development of a robust personalized intervention strategy that leverages technology in the unsupervised setting to foster lasting behavior change in stroke survivors. Future work should assess whether feedback provided directly on the activity watch, by email or a companion app is effective for maximizing functional movement behavior.

## Figures and Tables

**Figure 2:**
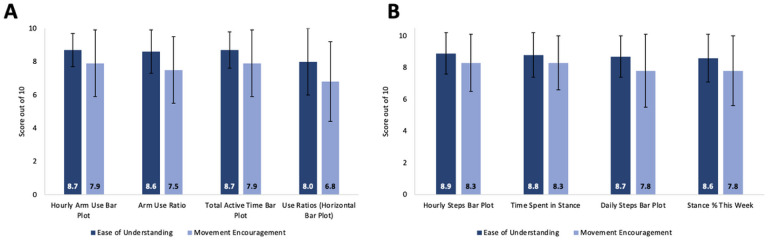
Rating of ease of understanding and movement encouragement of each feedback metric: The average scores were rated out of 10 for ease of understanding and movement encouragement from the visual analogue scales on the Movement report survey. Ease of understanding scores are in dark blue, and movement encouragement scores are in light blue for each (A) arm/hand and (B) mobility metric.

**Table 1 –:** Participant characteristics

Characteristic	Mean ± SD or %
Gender (%)	Men: 60.0
	Women: 36.7
	Non-binary: 3.3
Age (years)	58.6 ± 13.1
Race (%)	American Indian or Alaska Native: 0
	Asian: 16.7
	Black: 16.7
	Native Hawaiian or Pacific Islander: 10
	White or Caucasian: 36.7
	More than one Race: 16.7
	Not reported/unknown: 3.3
Ethnicity (%)	Hispanic: 30.0
	Non-Hispanic: 70.0
Time since stroke (years)	7.6 ±4.5 (range: 1.0–21.2)
Hemisphere affected by the stroke (%)	Left: 60.0
	Right: 40.0
Stroke classification (%)	Hemorrhagic: 23.3
	Ischemic: 70.0
	Not reported: 6.7
Limb concordance (%)	63.3
National Institutes of Health Stroke Scale (/42)	2.8 ± 2.0
Montreal Cognitive Assessment (/30)	24.7 ±3.4
Fugl-Meyer Assessment Upper Extremity (/66)	41.2 ±18.0 (Range: 18–66)
Functional Ambulation Category (%)	3: 13.3
	4: 26.7
	5: 60.0
10-Meter Walk Test (m/s)	Self-paced: 0.75 ± 0.40
	Fast paced: 0.96 ± 0.53

## Data Availability

The datasets used and/or analysed during the current study are available from the corresponding author on reasonable request.

## Abbreviations

App: application
FAC: Functional Ambulation Category
FMA-UE: Fugl-Meyer Assessment Upper Extremity
